# Cytotoxic T lymphocyte responses against melanocytes and melanoma

**DOI:** 10.1186/1479-5876-9-122

**Published:** 2011-07-27

**Authors:** Gwendolen Y Chang, Holbrook E Kohrt, Tor B Stuge, Erich J Schwartz, Jeffrey S Weber, Peter P Lee

**Affiliations:** 1 Department of Medicine, Division of Hematology, Stanford University School of Medicine, Stanford, California, USA; 2 Department of Pathology, Stanford University School of Medicine, Stanford, California, USA; 3 Moffitt Cancer Center, Tampa, Florida, USA

## Abstract

**Background:**

Vitiligo is a common toxicity associated with immunotherapy for melanoma. Cytotoxic T lymphocytes (CTLs) against melanoma commonly target melanoma-associated antigens (MAAs) which are also expressed by melanocytes. To uncouple vitiligo from melanoma destruction, it is important to understand if CTLs can respond against melanoma and melanocytes at different levels.

**Methods:**

To understand the dichotomous role of MAA-specific CTL, we characterized the functional reactivities of established CTL clones directed to MAAs against melanoma and melanocyte cell lines.

**Results:**

CTL clones generated from melanoma patients were capable of eliciting MHC-restricted, MAA-specific lysis against melanocyte cell lines as well as melanoma cells. Among the tested HLA-A*0201-restricted CTL clones, melanocytes evoked equal to slightly higher degranulation and cytolytic responses as compared to melanoma cells. Moreover, MAA-specific T cells from vaccinated patients responded directly ex vivo to melanoma and melanocytes. Melanoma cells express slightly higher levels of MART-1 and gp100 than melanocytes as measured by quantitative reverse-transcriptase polymerase chain reaction (qRT-PCR) and immunohistochemistry.

**Conclusions:**

Our data suggest that CTLs respond to melanoma and melanocytes equally in vitro and directly ex vivo.

## Introduction

Recent FDA approval of ipilimumab for metastatic melanoma provides strong support for the ability of the immune system to mediate a beneficial effect against this disease. However, immunotherapies for melanoma, including ipilimumab [1] and adoptive cellular therapies [2], come with substantial toxicities, including vitiligo [3-5], ocular [6] and systemic autoimmunity [1]. As such, a major need in next-generation melanoma immunotherapy is to uncouple tumor immunity from autoimmunity [7]. To improve the functional effectiveness of melanoma-reactive CTLs, understanding the factors leading to recognition of self and the barriers to breaking immune tolerance is crucial.

Two decades ago, pioneering work from the Rosenberg [8] and Boon [9] groups first demonstrated that T cells infiltrating human melanoma often target self, non-mutated proteins that are also expressed by normal melanocytes. These include enzymes in the biosynthesis of melanin, such as MART-1, gp100, and tyrosinase [10]. How these self tumor-associated antigens (TAAs) elicit T cell responses in the context of melanoma remains unclear. It is suggested that TAAs are overexpressed in melanoma cells, thus eliciting responses by low avidity TAA-specific T cells that escape central deletion [11,12]. If true, this offers an opportunity to target melanoma without harming normal melanocytes by specifically eliciting low avidity TAA-specific T cells [13].

In this study, we address whether CTLs respond to and target melanoma cells and normal melanocytes differently. We utilized a set of MART- or gp100-specific CTL clones that were determined to be high, intermediate, or low avidity (recognition efficiency, RE) based on peptide titrations. We assessed both CTL degranulation via mobilization of CD107, an integral membrane protein within cytolytic granules [14-16], and target cell killing via chromium release assays. We also determined if target cells express the cognate TAAs at similar levels, and relate these to cytotoxicity.

## Materials and methods

### Effector Cells

CTL clones were generated using protocols as previously described [17]. Briefly, samples were obtained from four different patients (the patients were anonymously identified by numbers as "476", "422", "462", "520") with resected stage III or IV melanoma patients under informed consent approved by the institutional review boards of the National Cancer Institute (NCI; Bethesda, Maryland) and the Los Angeles County/University of Southern California; sample analysis was performed under protocols approved by the institutional review board of Stanford University. Peripheral blood mononuclear cell (PBMC) samples were obtained from patients after vaccination with melanoma-associated antigens (MAA) peptides MART 26-35 (27L) (ELAGIGILTV) and gp100 209-217 (210M) (IMDQVPSFV) at the University of Southern California Norris Cancer Center (Los Angeles, California). The samples were analyzed by FACS for MAA-specific T cells using HLA-A*0201/peptide tetramer-phycoerythrin (PE) made with MART A26 or gp100 209-217 (Beckman Coulter). Recognition efficiency and cytolytic capability of each CTL clone was determined as previously described [15,17].

### Target Cells

Melanoma cell lines Malme-3M, MeWo, A375 and the T2 cell line were purchased from American Type Culture Collection (ATCC, Manassas, Virginia), and mel526 was obtained from the Surgery Branch of NCI. Melanocyte line HeMn-MP 4C0197 was purchased from Cascade Biologics (Portland, Oregon), and lines HeMn-LP and HeMn-MP with lot numbers 3C0523, 3C0527, 3C0651, 3C0659, 3C0764, and 3C0661 were kindly provided by Dr. Gary Shipley (Cascade Biologics). HLA-A*0201 status was tested in each melanocyte lot using direct PCR by the Stanford Histocompatibility Laboratory (Stanford, CA). T2 cells were pulsed and washed with either one of the MAA peptides, MART 26-35 or gp100 209-217, at a concentration of 10 μg/mL for 1 hour in 7% CO_2 _prior to each assay.

### CD107 Mobilization Assay

All assays were done in duplicates with an effector to target (E:T) ratio of 1:1, 2 × 10^5 ^of CTLs and 2 × 10^5 ^target cells in each well of 96-well plates. T2 cells were prepared as described above. The following was added each well in order: 1 μl of 2 mM monensin (Sigma, St. Louis, Missouri) in 100% EtOH, 100 μl of target cells, 100 μl of effector cells and 1 μl each of CD107a-allophycocyanin (APC) and CD107b-APC antibodies (Abs). The cells are mixed well using a multichannel pipettor and brought into contact by centrifugation at 1000 rpm for 1 min. Effectors and targets were incubated at 37°C in 7% CO_2 _for 4 hours. After the incubation, the plates were centrifuged at 1100 rpm for 1 min to pellet cells, and the supernatant was removed. Cell-cell conjugates were disrupted by washing the cells using 1 x PBS with 0.02% sodium azide and 0.5 mM EDTA.

### Flow Cytometric Analysis

After incubation with CD107 Abs, cells were washed and further stained with anti-human CD8-FITC (Caltag Laboratories, Burlingame, California; dilution of 1:200) and CD19-CyChrome (Becton Dickinson, San Jose, CA; dilution of 1:80). Cells were incubated for 1 hour at 4°C and were washed twice before analysis. Cells were analyzed using a two-laser, four-color FACSCalibur (Becton Dickinson). A minimum of 30,000 events were acquired and analyzed using Flowjo (TreeStar, San Carlos, California). Lymphocytes were identified by forward and side scatter signals, then selected for CD8 positivity and CD19 negativity. Gated cells were plotted for CD107 verses CD8 to determine level of T cell degranulation. Gates were analyzed for number and percentage of cells.

### Chromium Release Cytotoxicity Assay and Determination of Recognition Efficiency

Cytotoxicity was measured in a standard ^51^Cr release assay and all experiments were done in triplicates for each condition. Briefly, target cells were labeled with ^51^Cr for overnight at 37°C in 7% CO_2_. T2 cells were pulsed with peptides in conditions described above. Effectors were incubated with targets at a ratio of 10:1 (E:T) for 4 hours, and chromium release was measured. Percent cytotoxicity was calculated using the mean of the triplicates. Cytotoxicity of each CTL clone is expressed by % specific lysis ± % std dev. To determine the recognition efficiency (RE), chromium-labeled T2 targets were pulsed with a range of native peptide concentrations, generally starting at 10^-6 ^M and decreasing by log steps to 10^-14 ^M. For each CTL clone, percent cytotoxicity was plotted against peptide concentration and the negative log of the concentration. The peptide concentration at which the curve crossed 40% cytotoxicity was recorded as the RE of that clone. All assays were done twice.

### Quantitative Reverse-transcriptase Polymerase Chain Reaction (qRT-PCR)

RNA from melanocytes, melanoma cells and unpulsed T2 were extracted as previously described [18]. cDNA synthesis was performed according to the manufacturer's protocol using Superscipt II reverse transcriptase (Invitrogen, Carlsbad, California) primed with oligo-dT. Oligonucleotide primers used in qRT-PCR were synthesized based on published MART-1 and gp100 primer sequences [19]. Both primers were synthesized commercially by Elim Biopharmaceuticals (Hayward, California); the primer sequences are as follows: gp100(S): 5'-AGTTCTAGGGGGCCCAGTGTCT-3', (AS): 5'-GGGCCAGGCTCCAGGTAAGTAT-3'; MART-1 (Melan-A)(S):5'-TGACCCTACAAGATGCCAAGAG-3', (AS): 5'-ATCATGCATTGCAACATTTATTGATGGAG-3'. The real-time qRT-PCR was performed in single wells of a 96-well plate (BioRad, Hercules, California) in a 25 μl reaction mixture using components of the Sybr Green qPCR system according to manufacturer's protocol (Invitrogen). Cycling of cDNA involved denaturation at 95°C for 30s, annealing at 50°C for 1 min and extension at 70°C for 1 min for 40 cycles using the iCycler iQ^™ ^(BioRad). Fluorescence was measured following each cycle and displayed graphically (iCycler iQ Real-time Detection System Software, version 2.3, BioRad). The software determined a cycle threshold (Ct) value, which identified the first cycle at which the fluorescence was detected above the baseline for that sample or standard. The Ct value of MAA divided by Ct value of glyceraldehyde-3-phosphate dehydrogenase, an internal control, to express the relative ratio of mRNA expression in each cell line. Each qRT-PCR was performed in duplicate and data represents the mean of the duplicate of relative ratio in each condition.

### Immunohistochemistry

Formalin-fixed paraffin-embedded sections were obtained from primary or metastatic tumors and surrounding skin biopsies of patients with malignant melanoma in accordance with protocols approved by Stanford University. Monoclonal antibodies to Melan-A and gp100 (HMB45) were purchased from DAKO (Carpinteria, CA) and immunohistochemistry was carried out following the manufacturer's recommended conditions. Samples were analyzed in the Department of Pathology by a single pathologist (EJS). The extent of staining was scored as percentage of melanocytes or malignant cells testing positive for the presence of either Melan-A or gp100. Each patient sample was then assigned to one of three groups: <5%, 5-20%, >20%.

### Statistical analysis

Data are presented as mean ± standard error of mean. Two-tailed Student's T-test was used where appropriate with significance defined at p < 0.05. Standard linear regression analysis was used to determine correlation between degranulation and cytotoxicity assays.

## Results

### HLA-A2 Characterization of Target Cells and Recognition Efficiencies of Effector Cells

HLA-A*0201 status of each melanocyte cell line was analyzed using PCR-based analysis (Table 1). Melanocyte lines 4C0197 and 3C0661 are HLA-A*0201-positive, while 3C0659 expresses two different alleles (HLA-A*0202/0263) and 3C0764 is HLA-A2 negative. Melanoma lines Malme-3M, mel526, and MeWo are HLA-A*0201-positive and express MAAs gp100, MART-1, and tyrosinase. A375 is also a HLA-A*0201-positive melanoma line but is defective in intracellular processing and MHC presentation of gp100, MART-1, and tyrosinase [20]. MART-1 and gp100 specific CTL clones were previously isolated from PBMC samples of four post-vaccinated melanoma patients [15-17]. Antigen specificity and recognition efficiency (RE) of each clone are summarized in Table 2.

**Table 1:** Summary of HLA-A2 status in neonatal melanocyte lines

Melanocyte Line	HLA-A2 status
3C0651	(-negative)

3C0659	(-positive) A2*0202 (-positive) A2*0263

3C0764	(-negative)

3C0661	(-positive) A2*0201

4C0197	(-positive) A2*0201

**Table 2:** Characterization of MART-1 and gp100 - specific CTL clones by recognition efficiency.

MAA specificity	Clone	RE for native peptide (-log of peptide concentration, M)	Functional Avidity
**gp100**	476.140	11.2	high
	
	422.50	10.4	intermediate
	
	476.105	8.3	low

**MART-1**	461.24	7.7	high
	
	520.18	7.2	intermediate
	
	520.31	5.1	low

### CTL Degranulation Upon Contact with Melanocytes Compared to Melanoma Cells

**Figure 1:**
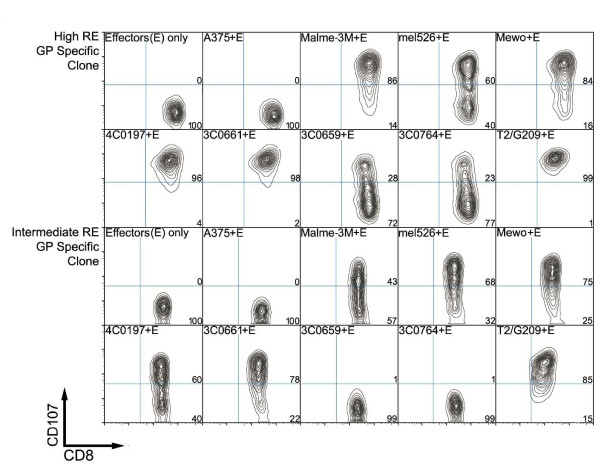
Representative FACS plot showing degranulation in HLA-A*0201-restricted gp100-specific CTL clones. CD107 mobilization quantification in gp100-specific, (A) high RE, and (B) intermediate RE CTL clones upon activation by target melanoma and melanocyte lines. CTL clones demonstrated MHC-restricted, peptide specific response against target cells with RE corresponding to levels as previously described [17]. All melanoma cell lines are HLA-A*0201-positive; melanocyte lines 4C0197 and 3C0661 are A*0201-positive while 3C0659 and 3C074 are A*0201-negative.

To examine CTL degranulation in the presence of melanocyte or melanoma cells, flow cytometric quantification of surface mobilization of CD107, an integral membrane protein in cytolytic granules, was employed using previously established protocol [14-17]. Functional reactivities of gp100 and MART-1 specific CTL clones in the presence of melanocyte lines HEMn-4C0197, 3C0661, 3C0659, and 3C0764 were compared with that in presence of melanoma lines A375, mel526, and Malme-3M using the CD107 degranulation assay. Two representative CD107 mobilization FACS assays are plotted in Figure 1, showing CTL degranulation of a high RE and an intermediate RE gp100-specific clone (Figure 1).

**Figure 2:**
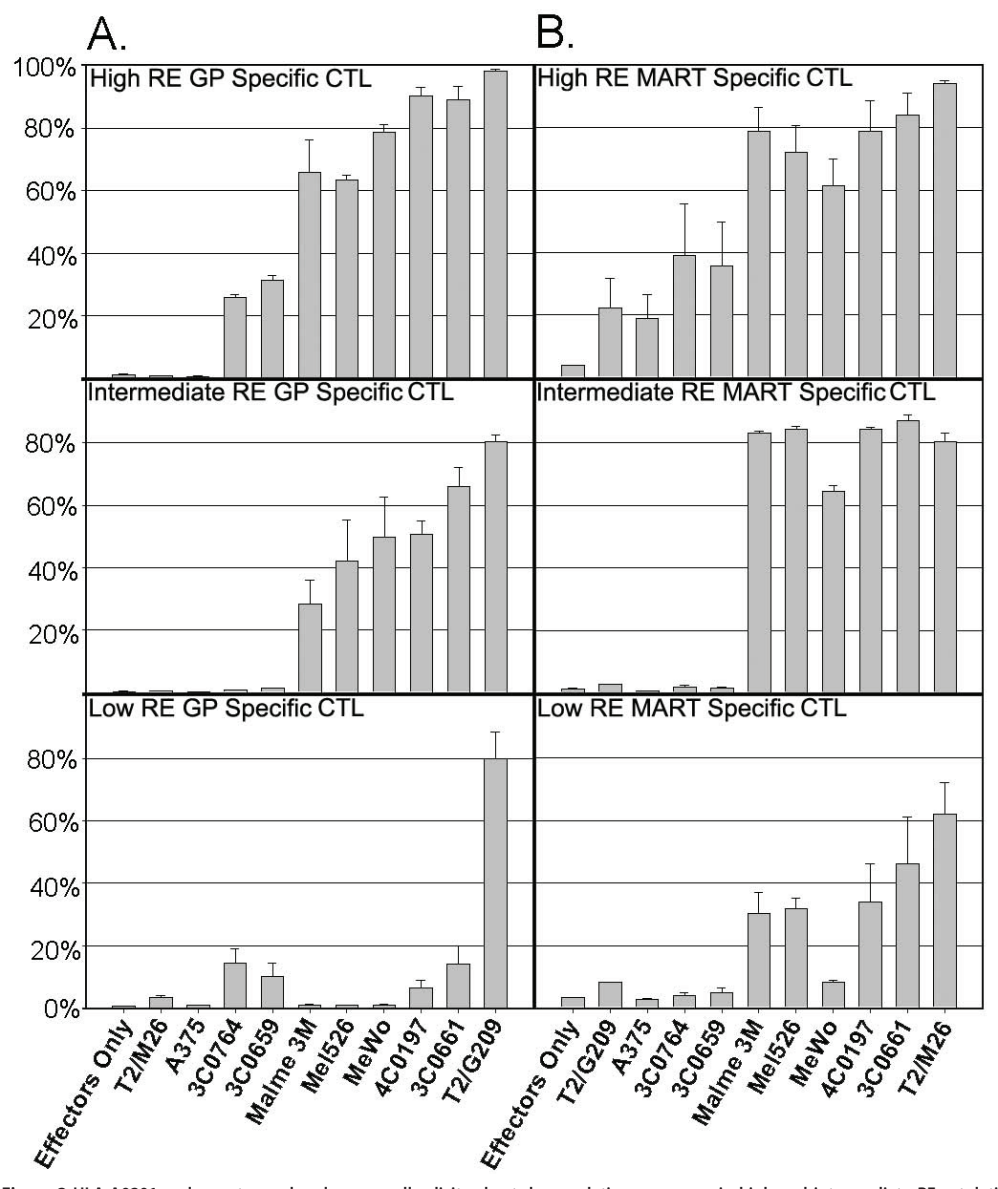
**HLA-A0201 melanocytes and melanoma cells elicit robust degranulation responses in high and intermediate RE cytolytic T cells**. (A) gp100-specific or (B) MART-1-specific CTL clones previously characterized as low, intermediate, or high RE [15,17] were incubated with various lines of melanoma, melanocyte and peptide-pulsed T2 cells for 4 hours. Lymphocytes were gated for CD8-positive cells and % population plotted for CD107-positivity was scored and plotted against each target cell line.

Mean percent degranulation of six tested clones, three gp100-specific (A) and three MART-1-specific (B), of high, intermediate or low RE, are plotted against each target cell line in Figure 2. For the high RE, gp100-specific CTL clone, degranulation was ~90% to both A2-positive melanocyte lines, versus 60-80% to melanoma lines Malme-3M, mel526, and MeWo (Figure 2A). This represents a modest but significant difference (p = 0.02). Both MART-1 and gp100-specific CTL clones of high avidity demonstrated a moderate level (25-39%) of CD107 degranulation against 3C0764 (HLA-A2 negative) and 3C0659 (HLA-A*0202/0263) melanocyte lines (Figure 2A and 2B, top panels). For the other clones, degranulation to A2-positive melanocytes and melanoma cells were to similar levels, with trends toward slight increases against melanocytes than melanoma (p = 0.1-0.15).

### Lymphocytes From Vaccinated Patients Are Reactive Against Melanocytes Ex Vivo

**Figure 3:**
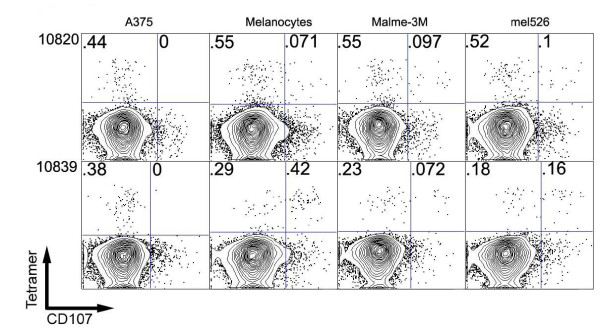
**Degranulation responses in ex vivo PBMC samples from peptide-vaccinated melanoma patients against melanocyte and melanoma cell lines**. PBMC samples were collected from two post-vaccinated melanoma patients (patient identification numbers 10820 and 10839). FACS plots demonstrating CD107 versus CD8 levels in the two patient samples after contact with the target cell lines. CD8-positive cells were further gated, showing percentage of CTLs staining positive for CD107 mobilization.

Two PBMC samples isolated from peptide-vaccinated patients were tested and found to be capable of eliciting HLA-/MAA-specific degranulation against both HLA-A*0201-positive melanocytes and melanoma directly ex vivo (Figure 3). Of CD8+ T cells, 0.2-0.5% were gp100 pMHC tetramer-positive (Figure 3). Amongst pMHC tetramer+ CD8+ T cells isolated from patient 10820, 0% degranulated against antigen-deficient melanoma A375, 11% degranulated against A*0201-positive melanocytes, 15% and 16% degranulated against melanoma lines Malme3M and mel526. For patient 10839, 1%, 59%, 24%, and 47% of CD8+ tetramer+ T cells degranulated against A375, A2-positive melanocytes, Malme3M, and mel526, respectively. These results suggest that peripheral blood CTLs from vaccinated patients are reactive against both melanoma and melanocytes directly ex vivo, at similar extents.

### Melanocytes are Equally Prone To CTL-Mediated Lysis as Melanoma Cells

**Figure 4:**
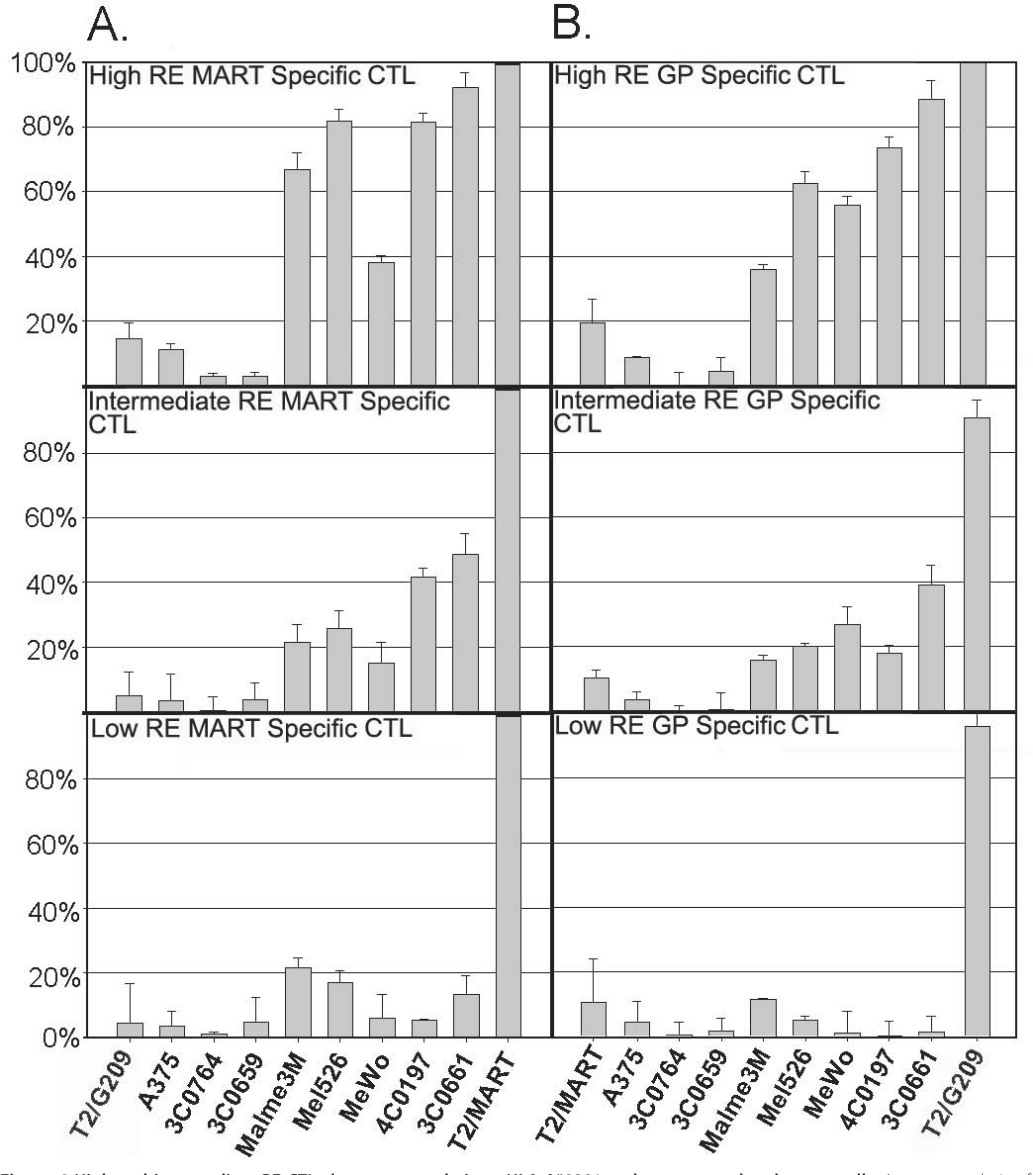
High and intermediate RE CTL clones are cytolytic to HLA-A*0201 melanocytes and melanoma cells. Average cytolysis of melanoma, melanocyte, and T2 targets by high, intermediate, or low RE MART- (A) or gp100-specific (B) CTL clones. Cytotoxicity of each CTL clone is expressed by % specific lysis ± % std dev. All assays were done in triplicates and repeated.

All CTL clones were functional and specific as demonstrated by lysis of T2 cells presenting relevant or irrelevant peptides (Figure 4). CTL lysis was HLA-restricted and antigen-specific, as HLA-A2 unmatched melanocytes and antigen-deficient melanoma line A375 had low cytotoxicity, ranging from 0-10%. For MART-specific clones, cytotoxicity reached 80-90% against A*0201-positive melanocyte lines compared to 40-80% against A2-positive melanoma lines by high RE clones (p = 0.19), and 40-50% against melanocytes versus 15-25% against melanoma cells by intermediate RE clones (p = 0.02). For gp100-specific clones, cytotoxicity was 70-90% against melanocytes versus 35-60% against melanoma (p = 0.08) by high RE clones, and 18-40% against melanocytes versus 15-25% against melanoma cell lines (p = 0.6) by intermediate RE clones. Low RE clones had little to no cytotoxicity (<20%) against melanoma or melanocytes, even though they had robust (95-100%) lysis against T2 pulsed with the relevant peptide. These data represent a modest but not statistically significant increase in CTL-mediated lysis of melanocytes compared to melanoma, with the exception of the intermediate RE, MART-specific clone. A robust correlation (r^2 ^= 0.80-0.88) was shown to exist between the degree of cytolytic activity and degranulation against various target cells, consistent with our previous results establishing CD107 mobilization as both an indicator of functional RE and target susceptibility [15,17,21].

### Quantification and Comparison of Melanoma-Associated Antigen Expression In Melanocytes Versus Melanoma Cells

To examine if an increased level of MAA expression underlies the strength of CTL-target interaction, we employed qRT-PCR in examining whether the amount of MAA mRNA may correlate with the extent of CTL degranulation and cytotoxicity. A minor difference was seen between the levels of MART-1 and gp100 mRNA expression in melanocyte and melanoma cells (Table 3). In HLA-A2-positive melanoma cells, MART-1 expression is 1.23-fold and gp100 expression is 1.11-fold higher than those expressed in A*0201-positive melanocytes (p < 0.015). In addition, skin biopsies from melanoma patients were analyzed by a semi-quantitative approach to characterize surface MAA presentation in both benign and malignant tissue. As shown in Table 4, expression of both MART-1 and gp100 was variable in each of the samples. However, 3 out of the 5 samples (Cases 2, 3, and 5) expressed comparable amounts of MAAs in both melanocyte and melanoma clusters. In most cases (Cases 2-5), >20% of both melanocytes and melanoma cells expressed MART-1.

**Table 3:** Relative ratio of TAA mRNA expression in each target cell compared to glyceraldehye-3-phosphate dehydrogenase.

Target Cell Antigen	A375	Malme3M	MeWo	Mel526	4C0197	3C0661	3C0659	3C0764	T2	water
**MART-1**	0	1.225	1.21	1.315	1.035	1.06	1.085	1.08	0	0

**gp100**	2.295	1.285	1.225	1.3	1.195	1.115	1.23	1.175	0	0

**GAPDH**	1	1	1	1	1	1	1	1	1	0

**Table 4:** Immunohistochemistry staining for MART and gp100 in melanoma and melanocyte clusters in 5 melanoma patient cases.^#^

Case	Diagnosis	MelanA			gp-100 (HMB45)		
		20%	5-20%	<5%	> 20%	5-20%	<5%
**1**	NA						
	
	Melanoma (SS)	*					*

**2**	IDN	*					*
	
	Melanoma (recur)	*					*

**3**	JMN	*			*		
	
	Melanoma (SS)	*			*		

**4**	IDN	*					*
	
	Melanoma (SS)	*			*		

**5**	IDN	*					*
	
	Melanoma (Nevoid)	*					*

^**#**^samples are scored based on percentage of melanocytes or malignant cells which stained histologically positive for either MelanA (MART-1) or gp100 in a given skin sample.

## Discussion

Autoimmunity against melanocytes has been observed to correlate with better clinical outcomes in malignant melanoma patients both anecdotally and in clinical trials of immunotherapies [8,11,22-25]. Can this treatment-related toxicity be uncoupled from anti-tumor activity? In this study, to examine the association between tumor killing and autoimmunity, MAA-specific CTLs were tested for degranulation and cytolysis against melanocyte and melanoma targets. MART-1 and gp100-specific CTL clones of high RE responded against melanocytes and melanoma targets, with a trend toward higher reactivity against melanocytes than melanoma. High avidity HLA-A*0201-specific clones non-specifically degranulate against A*0201-negative melanocyte lines at low levels insufficient for killing.

To address the notion that melanoma cells overexpress MAAs and may be preferentially targeted by lower RE CTLs that escape thymic deletion, we also analyzed reactivity patterns of intermediate and low RE CTL clones. Intermediate RE, MAA-specific CTLs responded comparably or slightly higher against melanocytes than melanoma cells. Low RE, MAA-specific CTLs showed little to no response against melanocytes and melanoma cells, even though they robustly lysed T2 cells pulsed with relevant peptide. Thus, these data argue against a previously held notion that low RE, MAA-specific CTLs can preferentially target melanoma cells and not normal melanocytes. Rather, these data suggest that MAA-specific CTLs respond against melanoma and melanocytes equally in vitro. This is consistent with a study showing melanoma lysis by vitiligo lesion-infiltrating CTLs [26]. This is not limited to in vitro expanded CTL clones, but also in directly ex vivo CTLs from patients post-vaccination. Technical challenges imposed by limited patient samples and low proportions of tumor-specific CTLs in the PBMC do not allow for a more detailed analysis or direct comparison to our in vitro observations. However, by selecting pMHC tetramer+, CD8+ T cells which represent MART-1 or gp100-specific CTLs, we observed similar levels of degranulation from these ex vivo CTLs upon contact with HLA-A2 melanocytes as compared to HLA-A2 melanoma cells.

In this study, there is a trend towards a lower degranulation efficiency of MART-1 specific clones against T2 target cells pulsed with MART peptides, when compared to gp100-specific clones against T2 pulsed gp100 peptides. In our previous studies, the RE scores observed for MART-1 specific clones presented with MART peptides were at a relatively lower range compared to clones presented with other peptides [15-17]. We hypothesize that this is likely due to the short predicted half-life of MART peptides (native and heteroclitic) in complex with the HLA-A*0201 molecule. Moreover, Rubio-Godoy et al. [27] found discrepancy between CTL effector functions measured by cytokine secretion and target cell lytic activities in their tyrosinase-specific clones. In their study, T cell clones detected by IFN-γ ELISPOT but not detectable by pMHC multimer staining were able to lyse tyrosinase peptide-pulsed target cells as efficiently as those stained by pMHC multimers. The authors attributed such differences to the kinetics of pMHC-multimer interaction with TCR among the clones studied. We speculate that while the lower degranulation efficiency correlates to the low RE observed for our MART-1 specific clone as expected, the high cytotoxicity observed may be a reflection of co-stimulation of other cytokine production such as IFN-γ following CD107 degranulation.

Vaccine immunotherapy for melanoma can be associated with autoimmune effects of vitiligo. The incidence of vitiligo in patients with melanoma, although rare, is estimated to be seven to ten-fold higher than the general population [28]. The occurrence of vitiligo in melanoma patients undergoing immunotherapy may be due to both qualitative and quantitative differences between the CD8+ T cells in the two diseases. In a murine model by Steitz et al. [29], there appeared to be a two-step requirement for MAA-specific CD8+ T cells to break tolerance in the development of vitiligo. First, the stimulation and expansion of MAA-specific CD8+ T cells requires CD4+ T cell help in vivo during the "induction phase". Then, in the "effector phase", the CD8+ T cells require a strong local inflammatory stimulus for autoimmune destruction of melanocytes within the skin. Garbelli et al. [4] also reviewed data supportive of a qualitative difference between MAA-specific T cell responses in vitiligo and melanoma. In the several studies reviewed, CD8+ T cells isolated from vitiligo lesions or patients were found to have augmented functional avidity than those from their melanoma counterparts.

From a quantitative standpoint, incidence of vitiligo may be rare due to the low percentages of functional CTLs against melanoma antigens in the peripheral blood after vaccination. Our data is largely similar to what had been observed in other published studies. In study by Jacobs et al. [30], the authors found that when vitiligo occurs, MAA-specific CD8+ T cells were observed in high percentages in both tumor and vitiligo lesions, supportive of the hypothesis that vitiligo may not be uncoupled from anti-tumor effect, and even indicative of the success of immunotherapy. However, only <0.2% of the peripheral lymphocyte isolated from the studied patient demonstrated MAA-specific tetramer staining. In this study, <0.6% of peripheral blood lymphocytes from our post-vaccinated patient samples demonstrated MAA-specific activity.

It is suggested that target recognition by CD8 T cells is dependent upon a critical threshold amount of MHC/MAA peptide expression on the cell surface [31-33]. Studies have shown that MAA expression may be highly variable across various clinical stages and different melanoma samples [34-36], with tumor escape from immune recognition achieved by loss of MAA or MHC expression [36-40]. Our data suggest that melanocytes and melanoma cells express MAAs at or above the recognition thresholds of high RE CTLs, as these effectors lysed both targets equally even though melanoma cells express the relevant MAAs at slightly higher levels. In contrast, for intermediate and low RE CTLs, lysis of melanoma and melanocytes was substantially below lysis of T2 pulsed with excess peptide. As such, increasing MAA expression levels specifically in melanoma cells, in context of immunotherapy with intermediate and low RE CTLs may be a possible avenue to uncouple tumor immunity from autoimmunity.

## Conclusions

Among the tested HLA-A*0201-restricted CTL clones in this study, melanocytes evoked equal to slightly higher degranulation and cytolytic responses as compared to melanoma cells. Furthermore, MAA-specific T cells from vaccinated patients responded directly ex vivo to melanoma and melanocytes equally. These results suggest that CTL recognition and killing of melanoma may not be differentiated from autoimmune cytotoxicity of normal melanocytes.

## Competing interests

The authors declare that they have no competing interests.

## Authors' contributions

GYC carried out the biochemical studies, immunoassays, participated in the statistical analysis, discussion of results and drafted the manuscript. HEK carried out the immunoassays, participated in the discussion of results and drafted the manuscript. TBS coordinated the pre-testing experiments, contributed to the refinement of experiment protocol and participated in the discussion of results. EJS performed the immunohistochemistry. JSW selected the donors for the study. PPL conceived the study, participated in its design and coordination and drafted the manuscript. All authors read and approved the final manuscript.
